# Awake 160-Slice Computed Tomography for Upper Airway Evaluation in 17 Dogs

**DOI:** 10.3390/vetsci11080342

**Published:** 2024-07-29

**Authors:** Marius B. Stordalen, Sharyn Bray, Felicity Stringer, Callum Stonebrook, Sergio Guilherme, Jonathan P. Bray

**Affiliations:** AURA Veterinary LTD, Guildford GU2 7AJ, UKjonathanb@auravet.com (J.P.B.)

**Keywords:** awake computed tomography, upper airway disease, brachycephalic obstructive airway syndrome, dogs

## Abstract

**Simple Summary:**

This study investigated using a high-quality computed tomography (CT) scanner to aid the diagnosis of upper airway disease in dogs without the need for sedation or anaesthesia. Data from seventeen dogs with breathing problems were included, and were classified into three groups based on their condition and whether they had previously undergone airway surgery. Commonly reported clinical signs included noisy breathing and difficulty exercising. Results showed this imaging technique may provide an efficient and safe technique to evaluate the anatomical structures of the upper airway. Understanding upper airway diseases better can improve treatment outcomes, especially for breeds prone to airway disease, laying a foundation for further advancements in veterinary care.

**Abstract:**

This study investigated using a 160-slice multidetector computed tomography (CT) scanner for conscious image acquisition in dogs with upper airway disease, and describes findings in dogs that had previously undergone soft palate surgery. Seventeen client-owned dogs with upper airway disease were retrospectively reviewed, and classified into three groups: group I, “untreated brachycephalic obstructive airway syndrome (BOAS) patients”; group II, “previously treated BOAS patients”; and group III, “patients with respiratory disease other than BOAS”. Data included signalment, clinical history, direct laryngoscopy and endoscopy findings, previous surgeries and CT findings. CT scans in group I revealed overlong and thickened soft palates in all dogs, and signs of laryngeal collapse in four dogs. Patients in group II exhibited normal soft palate lengths, while patients in group III displayed various findings such as nasopharyngeal narrowing and tracheal collapse. Upper airway examinations under general anaesthesia confirmed most CT findings. This study demonstrates the feasibility and value of conscious CT scanning for assessing upper airway diseases in dogs, providing valuable diagnostic information while eliminating the need for chemical immobilisation, thus reducing patient stress and cost. These findings offer new insight into upper airway anatomy in awake patients, especially in brachycephalic breeds, and lay a foundation for future research.

## 1. Introduction

The upper airways include the nares, nasal passages, pharynx and larynx. Some authors also include the trachea [1]. Due to the complex anatomy of the upper airways, advanced imaging modalities are often used during the investigation of upper airway diseases [2,3]. Computed tomography (CT) has been explored as a valuable tool for assessing upper airway dimensions [2,3,4,5,6,7,8,9,10]. 

Traditionally, chemical immobilisation by means of general anaesthesia or sedation is employed during image acquisition to ensure adequate positioning of the patient and to limit motion artifacts [3,11]. The precise impact of chemical immobilisation on upper airway dimensions remains unknown at present. It is plausible that alterations in the muscular tone of the soft tissues within the upper airways may influence the recorded dimensions and anatomical relationships during CT investigations. 

While CT examinations have been conducted in awake dogs and cats, these studies have been limited to scanners with fewer than 16 slices, with a predominant focus on thoracic and abdominal imaging [12,13,14,15,16,17,18,19,20,21,22]. A study by Stadler and O’Brien described awake CT scanning of the head, neck and thorax in 14 cats, utilising a transparent positioning device (VetMouseTrap, University of Illinois, Urbana, IL) during image acquisition [21]. Similar devices have been employed in other studies involving cats and dogs [12,17,18,20,22]. To the authors’ knowledge, no reports in the existing literature describe the use of conscious CT for the assessment of the pharynx and palate in awake dogs. Furthermore, there is a paucity in the literature regarding awake CT scans in patients who have previously undergone upper airway surgery. Lastly, the use of greater-than-16-slice CT scanners for conscious image acquisitions have not been documented. 

In light of these gaps in knowledge, the primary objective of this study was to describe the use of a 160-slice multidetector CT scanner in evaluating dogs with upper airway disease in a cohort of dogs undergoing image acquisition without the use of sedatives or general anaesthesia. A secondary objective was to report a cohort of dogs that had previously undergone soft palate surgery, using conscious CT scanning. 

## 2. Materials and Methods

### 2.1. Study Population

This retrospective study included client-owned dogs with complete clinical records, which were seen at the authors’ referral hospital between April 2020 and April 2023, and underwent conscious CT scans for assessment of upper airway disease. Cases where conscious CT scanning had been performed but had provided non-diagnostic CT images were also included. Cases where sedative or anaesthetic drugs were administered prior to, or during image acquisition were excluded from the study. Informed consent was obtained from all dog owners, including acceptance by the owners for their dog to participate in the current study. Ethical review and approval were waived for this study due to its retrospective nature.

### 2.2. Data Collection

Data collected included signalment, history prior to presentation, physical examination findings, upper airway examination findings, details of previous upper airway surgical procedures, endoscopy findings and CT findings. Cases were categorised into three groups, namely group I: “patients with untreated brachycephalic obstructive airway syndrome (BOAS)”; group II: “patients previously treated for BOAS; and group III: “patients with respiratory disease other than BOAS”.

### 2.3. Computed Tomography

**Figure 1:**
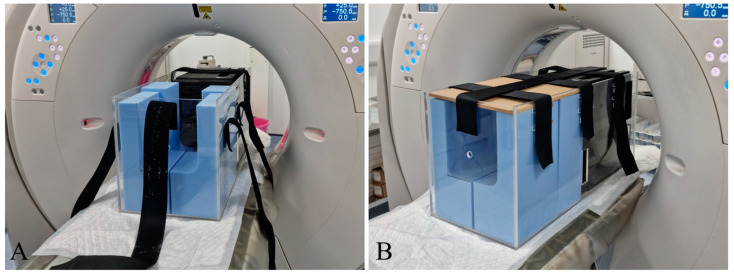
Positioning device. (**A**) Open position. (**B**) Top lid secured.

For conscious CT scans, a custom-built transparent acrylic, low-attenuating positioning device was designed and built, allowing dogs to be scanned while in a sternal or standing position (Figure 1). Prior to placing the dog in the positioning device, a pre-scan scout was conducted of the positioning device to minimise the time the patient was required to spend inside the device. Dogs were comfortably situated in the device in a sternal or standing position, with their head in a neutral position. Foam cushions were strategically placed within the device to encourage the dogs to help maintain them in a sternal or standing posture, and to limit movement of the head and body. A wooden lid, secured with Velcro tapes (Figure 1), prevented the foam cushions from shifting during the scan. A ventilation hole was incorporated into the front wall of the device to ensure proper airflow. Importantly, no sedative or general anaesthetic drugs were administered prior to or during image acquisitions. Dogs were given time to acclimatise within the positioning device prior to the image acquisition, and their head positions were continuously monitored by personnel situated in the CT control room. No staff members were in the CT room during image acquisition. If the patient was non-compliant and would not remain still in the box, no imaging was attempted, and the study was abandoned. If the patient settled, the scan was initiated once a stable head position was visually confirmed. Verbal and/or visual distractions were often employed by the personnel through the microphone and window of the CT control room to help maintain the dog’s focus to the front of the positioning device. The dogs were continuously visually monitored during image acquisitions for signs of swallowing, excessive tongue movements, yawning and panting. Any scans that exhibited severe motion artefacts, head turning, signs of swallowing, excessive tongue movements, yawning or panting were repeated. Up to two repeated scans (total of three scans) were performed in any dog, and the scans were considered unsuccessful if non-diagnostic images were not achieved within a total of three scans. The total image acquisition time was 3–5 s. The image quality was assessed both by a residency trained radiologist and a board-certified surgeon.

All dogs underwent a non-contrast CT scan of the head and neck using a 160-slice CT scanner (Toshiba Aquilion™ PRIME, Toshiba Medical Systems Corporation, Tokyo, Japan). In some cases, inclusion of the thoracic cavity was performed based on the clinician’s preference or if relevant clinical signs were present. CT images were acquired in a helical mode with the following settings: tube voltage: 120 kV; tube current: 150 mA; gantry rotation speed: 0.35 s; field of view: 40 cm; collimation: 80 rows x 0.5 mm; slice thickness: 0.5 mm; pitch factor: 1.388; table feed per rotation: 55.5 mm. Thin axial slices were reconstructed with a slice thickness of 1 mm and a slice interval of 0.8 mm using both soft tissue and bone algorithms. Multiplanar reconstructions were then created using Toshiba Aquilion Prime version 7 software (Toshiba Medical Systems Corporation, Tokyo, Japan) in the sagittal, dorsal and transverse planes with a slice thickness of 1 mm and slice reconstruction interval of 1 mm. Scans were conducted from a rostral to caudal direction. The dogs were promptly removed from the positioning device following successful image acquisition. All CT scans were viewed using commercially available software (Horos version 4.0.0 64-bit), using a window width of 400 Hounsfield Units, and window level of 40 Hounsfield Units. 

The soft palate length and thickness was subjectively assessed in the sagittal plane. The soft palate was considered overlong when the soft palate extended more than 2 mm beyond the dorsal border of the epiglottic cartilage [23].

The soft palate was classified as “markedly thickened” if the dorsal (nasopharyngeal) surface of the soft palate was deviating dorsally causing more than 75% narrowing of the nasopharynx, and also included cases were there was contact between the ventral and dorsal nasopharyngeal walls. The soft palate was classified as “moderately thickened” if the dorsal (nasopharyngeal) surface of the soft palate was deviating dorsally without contacting the dorsal nasopharyngeal wall, causing 25–75% narrowing of the nasopharynx. The soft palate was classified as “mildly thickened” if the dorsal (nasopharyngeal) surface of the soft palate was deviating dorsally without contacting the dorsal nasopharyngeal wall, causing less than 25% narrowing of the nasopharynx. The soft palate thickness was classified as “normal” if the dorsal (nasopharyngeal) surface of the soft palate was parallel to the dorsal nasopharyngeal wall, with no narrowing of the nasopharyngeal diameter. 

The cross-sectional dimensions of the nasopharynx, laryngeal glottis and trachea were specifically assessed in the transverse plane. The presence of tracheal hypoplasia was assessed using the ratio of tracheal diameter to thoracic inlet (TD:TI) as previously described by Harvey and Fink [24]. Tracheal hypoplasia was defined as a TD:TI <0.2 in non-brachycephalic dogs, <0.16 for non-English Bulldog brachycephalic dogs and <0.12 in English Bulldogs [24].

### 2.4. Upper Airway Examination

In patients where an upper airway examination was performed, this was performed upon induction of general anaesthesia in a varied time period after a conscious CT scan had been conducted. In patients with laryngeal collapse, the degree of collapse was staged according to the grading system by Leonard [25].

### 2.5. Statistical Analysis

Descriptive statistics are presented as mean ± SD for normally distributed variables or as median and range for non-normally distributed variables. The Shapiro‒Wilk W normality test was employed to assess for normal distribution in our data material.

## 3. Results

### 3.1. Signalment

A total of 17 dogs met the inclusion criteria. Breeds included French Bulldogs (*n* = 6), Pugs (*n* = 3), Cavalier King Charles Spaniels (*n* = 3), Pomeranian (*n* = 1), Chihuahua (*n* = 1), Cocker Spaniel (*n* = 1), Maltese (*n* = 1) and crossbreed (*n* = 1). The median age was 3.9 years (range 3 months–12.5 years), and the mean body weight was 9.2 kg (SD ± 3.9 kg). Four of the 17 dogs were entire males, six were neutered males, two were entire females and five were neutered females. 

### 3.2. History and Clinical Signs

A history of increased upper respiratory sounds was reported in 16 of 17 dogs. Eleven dogs had a history of dyspnoea. Episodes of cyanosis, collapse and sleep apnoea were reported in four, five and eight dogs, respectively. Exercise intolerance was reported in 12 dogs, and regurgitation and vomiting were reported in five and four dogs, respectively. Coughing and retching were reported in three and four dogs, respectively. Four dogs had displayed signs of reversed sneezing, and two dogs had a history of aspiration pneumonia. 

Among the 17 dogs, 11 were presented for investigations of BOAS (Table 1). Within this group, seven dogs had no previous history of multi-level airway surgery, and were included in group I. The remaining four dogs had previously undergone multi-level airway surgery (cases 1, 5, 14 and 17), and were included in group II. Of these four dogs, one dog had undergone vertical wedge rhinoplasty (case 1). Two dogs had undergone folded flap palatoplasty (cases 1 and 14). Two dogs had undergone staphylectomy. Of the latter two dogs, one dog had undergone two staphylectomy procedures (case 17), and one dog had undergone staphylectomy, followed by revision surgery to reduce the thickness of the soft palate by an incisional palatoplasty (case 5). Tonsillectomy had been performed in two dogs (cases 1 and 14). Of the four dogs with a previous history of multi-level airway surgery, two dogs presented following episodes of collapse (cases 1 and 17), one dog due to persistent upper respiratory symptoms (case 5), and one dog due to intermittent sleep apnoea (case 14). These four dogs underwent conscious CT scans 190 (case 1), 469 (case 14), 881 (case 5) and 2682 (case 17) days after their most recent multi-level airway surgeries.

Six of the 17 dogs were investigated for upper airway conditions unrelated to BOAS (Table 1) and were included in group III. Of these cases, two presented with suspected tracheal collapse (cases 2 and 16), one with sleep apnoea (case 8), one with reversed sneezing (case 13), one with nasopharyngeal narrowing and tracheal collapse (case 12) and one with nasopharyngeal narrowing (case 4). Three cases (cases 4, 12 and 16) had had a CT scan performed under general anaesthesia 156 (case 4), 2 (case 12) and 260 (case 16) days before undergoing the conscious CT scan. These initial scans had documented nasopharyngeal narrowing (case 4), nasopharyngeal narrowing and tracheal collapse (case 12) and tracheal collapse (case 16). 

### 3.3. Computed Tomography Findings

#### 3.3.1. Group I

**Figure 2:**
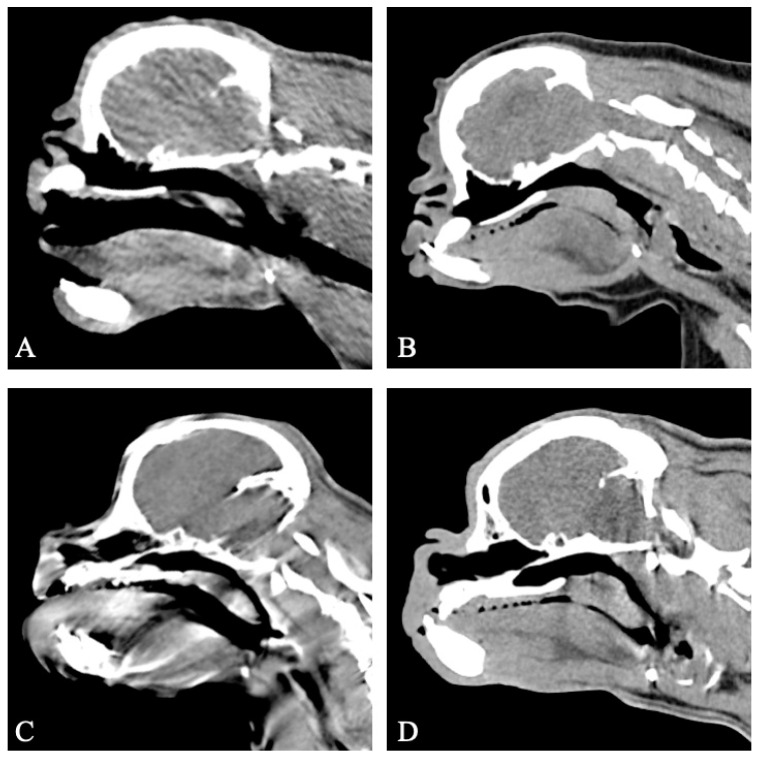
Sagittal non-contrast CT images of the skull of four patients with untreated brachycephalic obstructive airway syndrome (BOAS) (group I). All patients are displaying an overlong soft palate. All patients display varying degrees of soft palate thickening. (**A**) Case 6, mild soft palate thickening. (**B**) Case 3, moderate soft palate thickening. (**C**) Case 10, moderate soft palate thickening. (**D**) Case 9, marked soft palate thickening. Note the good visualisation of the soft palate despite motion artefacts in image C. Window width, 400 HU; window level 40 HU; 1 mm slice thickness.

The CT provided diagnostic images for all seven patients for the evaluation of the soft palate and nasopharynx, with no motion artifacts in five patients, and moderate motion artefacts in two patients. All seven dogs displayed an overlong soft palate. Thickening of the soft palate was assessed as mild (*n* = 1), moderate (*n* = 3), and marked (*n* = 3) based on the extent of nasopharyngeal obstruction (Figure 2).

**Figure 3:**
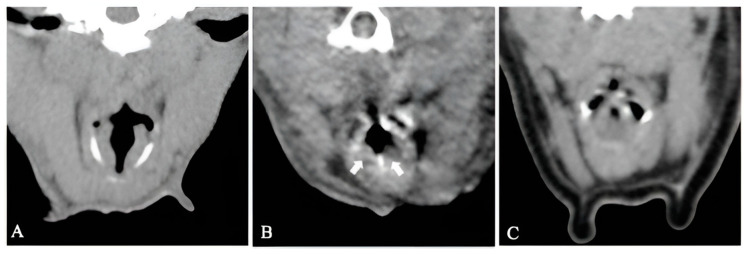
Transverse non-contrast CT images at the level of the larynx of patients with untreated BOAS (group I). (**A**) Case 7, no signs of laryngeal collapse. (**B**) Case 6, everted laryngeal saccules. (**C**) Case 3, marked laryngeal collapse. White arrows: everted laryngeal saccules. Window width, 400 HU; window level 40 HU; 1 mm slice thickness.

Tomographic appearance of laryngeal collapse was present in four of the seven dogs (Figure 3). Eversion of the laryngeal saccules was present in two dogs. Severe laryngeal collapse was present in two dogs (cases 3 and 15). The remaining three dogs had an open rima glottis.

Two dogs had caudal aberrant nasal turbinates present. Two dogs had evidence of unilateral otitis media, and diffuse oesophageal gas dilation was present in one dog. Tracheal hypoplasia was present in one French Bulldog, with a TD:TI of 0.13. Tracheal dimensions were normal for the breed in the remaining six dogs.

#### 3.3.2. Group II

**Figure 4:**
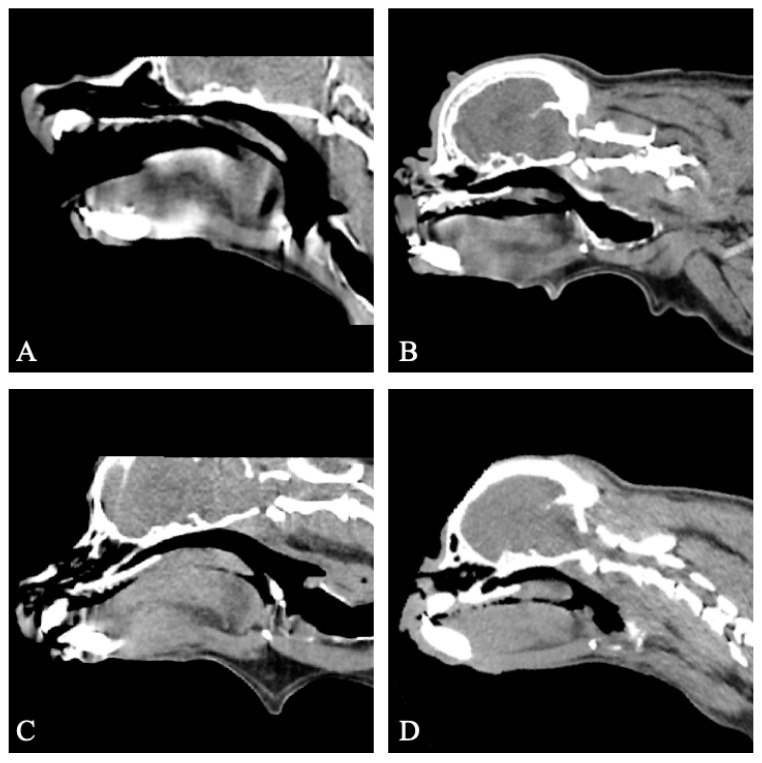
Sagittal non-contrast CT images of the skull of four patients previously treated for BOAS (group II). All patients have a normal soft palate length. All patients display varying degrees of soft palate thickening. (**A**) Case 5, normal soft palate thickness. (**B**) Case 17, mild soft palate thickening. (**C**) Case 14, mild soft palate thickening. (**D**) Case 1, moderate soft palate thickening. Window width, 400 HU; window level 40 HU; 1 mm slice thickness.

The CT provided diagnostic images for all four patients for the evaluation of the soft palate and nasopharynx, with mild motion artifacts in two patients. All of the four dogs had a soft palate of normal length (Figure 4). Thickening of the soft palate was assessed as normal (*n* = 1), mild (*n* = 2) and moderate (*n* = 1) based on the extent of nasopharyngeal obstruction (Figure 4).

Tomographic appearance of laryngeal collapse (eversion of the laryngeal saccules) was present in one of the four dogs (case 17). The remaining three dogs had an open rima glottis. In one dog, severe multifocal pulmonary soft tissue masses were present, compatible with advanced metastatic disease. A definitive primary tumour was not detected. Tracheal dimensions were normal in all dogs. 

#### 3.3.3. Group III

**Figure 5:**
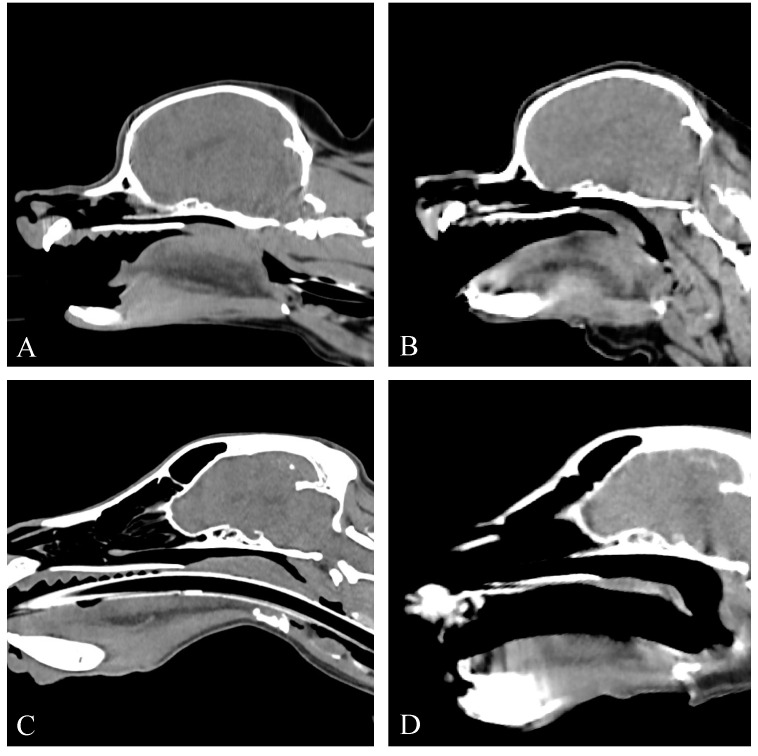
Sagittal non-contrast CT images of the skull of two patients assessed for nasopharyngeal narrowing (group III). (**A**) Case 12, CT under anaesthesia showing severe nasopharyngeal narrowing. (**B**) Case 12, conscious CT showing resolution of nasopharyngeal narrowing following steroid treatment. (**C**) Case 4, CT under anaesthesia showing moderate soft palate thickening and caudo-dorsal nasopharyngeal hypertrophy causing nasopharyngeal narrowing. (**D**) Case 4, conscious CT showing normal palate thickness and resolution of nasopharyngeal narrowing following steroid treatment. Window width, 400 HU; window level 40 HU; 1 mm slice thickness.

The CT provided diagnostic images for all six patients for the evaluation of the soft palate and nasopharynx, with mild motion artefacts in one patient, and moderate motion artifacts in one patient. Three of the six dogs had an overlong soft palate (cases 8, 12 and 13). Thickening of the soft palate was assessed as normal (*n* = 3) and mild (*n* = 3) based on the extent of nasopharyngeal obstruction. An open rima glottis was present in all six dogs. In one dog (case 12), severe nasopharyngeal narrowing was evident (Figure 5). At a subsequent CT scan following improvement in clinical signs following treatment with anti-inflammatory corticosteroids, normal nasopharyngeal dimensions were now present (Figure 5). While the head position appeared consistent between the two scans, and no motion artefact was detected during image acquisition to suggest swallowing, the potential for this to be artefactual cannot be fully discounted. In another dog (case 4), a prior CT scan conducted under general anaesthesia had revealed moderate soft palate thickening, causing nasopharyngeal narrowing. However, the conscious CT scan demonstrated a reduction in palate thickness and resolution of the nasopharyngeal narrowing (Figure 5). The patient had received anti-inflammatory corticosteroid treatment in the time period between the two scans. 

Tracheal collapse was present at the thoracic inlet in two dogs. One case had a moderate collapse (case 12), and one case had severe collapse (case 16). Tracheal dimensions were normal in the remaining four dogs. Unilateral otitis media was present in one dog. 

### 3.4. Upper Airway Examination

In total, nine of the seventeen dogs underwent an upper airway examination upon induction of general anaesthesia at a median of 6 days (range 0–25 days) following the conscious CT scan, either as part of further investigation or prior to surgical intervention. The findings are summarised in Table 1.

#### 3.4.1. Group I

Five of the seven patients with untreated BOAS had an upper airway examination performed. Among the five dogs in this group, all displayed an overlong and thickened soft palate. All these five dogs had an overlong and thickened soft palate documented on CT. 

Four patients had laryngeal collapse documented, with two dogs showing tomographic appearance of laryngeal collapse. Of these, three dogs had a stage 1 laryngeal collapse, with one of these dogs having tomographic appearance of everted laryngeal saccules. The remaining two dogs with stage 1 laryngeal collapse had no evidence of laryngeal collapse on CT. One dog had a stage 3 laryngeal collapse confirmed, which also had tomographic appearance of severe laryngeal collapse. Four of the seven dogs had everted palatine tonsils recorded.

#### 3.4.2. Group II

None of the four patients in this group underwent an upper airway examination following the conscious CT scan. 

#### 3.4.3. Group III

Four of the six dogs in this group underwent an upper airway examination. All four dogs had a normal length of their soft palate recorded. Two of these four dogs had tomographic appearance of an overlong soft palate, and the remaining two dogs had a normal palate length on CT. All four dogs had a normal soft palate thickness recorded, of which two dogs had a normal soft palate thickness, and two dogs had mild soft palate thickening on CT. 

None of the dogs had laryngeal collapse recorded, and all four dogs had an open rima glottis on CT. None of the four dogs had laryngeal paralysis recorded. Two of the four dogs had everted palatine tonsils recorded.

### 3.5. Endoscopy

Two dogs underwent endoscopic examination following their conscious CT scan (Table 1).

One case underwent nasopharyngoscopy (case 12), which confirmed resolution of nasopharyngeal narrowing. Two cases underwent tracheobronchoscopy. Grade 2 tracheal collapse was documented in one case (case 12), and grade 4 tracheal collapse was documented in one case (case 16).

## 4. Discussion

To the authors’ knowledge, this is the first study to retrospectively describe the use of a 160-slice CT scanner for conscious image acquisition for assessment of upper airway disease in a cohort of dogs. Similarly, this is the first study reporting conscious CT findings in dogs that had previously undergone multi-level airway surgery for BOAS. 

Conscious CT scanning represents a significant leap forward in diagnostic capabilities for evaluating upper airway disease in dogs. In this study, all 17 dogs had CT images of diagnostic quality, enabling the assessment of palate thickness and length, nasopharyngeal dimensions, signs of laryngeal collapse and tracheal dimensions. Several studies have described CT dimensions of the upper airways in anaesthetised dogs [2,3,4,5,6,7,8,9,10,26], however only two studies, to the best of our knowledge, have reported upper airway assessment in awake dogs using CT [15,20]. 

In one such study conducted by Lim and colleagues [15], tracheal shapes were characterised in 19 healthy dogs without any signs of respiratory malfunctions, using conscious CT scans. Notably, the dogs were positioned in lateral recumbency and physically restrained by being wrapped in a towel and taped on the CT table. Although it is unlikely that a lateral recumbency has any effect on the tracheal shapes in a population of normal dogs, it is unknown if lateral recumbency would have an impact on palatal or nasopharyngeal dimensions, or on the trachea in dogs with tracheal disease. In our study, all dogs were positioned in a sternal or standing position with a neutral head position, to avoid any potential influence of recumbency on upper airway dimensions. In the context of conscious CT scanning, the authors assert that the method of positioning dogs in lateral recumbency, utilising a combination of towel and tape for physical restraint, presents a noteworthy risk of inducing stress. This is particularly concerning for dogs with pre-existing respiratory conditions. The study from Lim and colleagues [15], which focused on healthy non-brachycephalic breeds, stands in contrast to our investigation. Our study included dogs exhibiting respiratory signs, with a notable proportion (12 out of 17) being brachycephalic breeds. We opted against physically restraining any dogs in lateral recumbency, deeming it a high-risk approach likely to exacerbate the pre-existing respiratory symptoms in our population. Instead, we employed a custom positioning device designed to facilitate a sternal or standing position, with the intention of mitigating stress levels during conscious CT scanning.

In another study by Stadler and colleagues [20], 15 of 17 included dogs underwent conscious CT and 3D internal rendering to assess non-neoplastic obstructions of the larynx, trachea or large bronchi. Nine dogs in this study had tomographic appearance of laryngeal collapse, however 3D internal rendering was reported most useful to stage the collapse. All nine dogs had laryngeal collapse confirmed by upper airway examination or necropsy. Palate or nasopharyngeal dimensions were not assessed in this study. In our population of dogs, one dog with confirmed stage 3 laryngeal collapse had tomographic appearance of laryngeal collapse, characterised by severe narrowing of the rima glottis. Of the three dogs in our population with confirmed stage 1 laryngeal collapse, only one dog had tomographic appearance of everted laryngeal saccules. These findings are in agreement with Stadler and colleagues’ findings [20], where use of 3D internal volume rendered images were required to provide accurate assessment of patients with laryngeal collapse. Three-dimensional internal volume rendered images were not used to assess our population of dogs, and this may explain why some of our patients with laryngeal collapse were missed. 

Ten dogs in our population had an overlong soft palate, and thirteen dogs had a thickened soft palate, on CT. Heidenreich and colleagues reported nasopharyngeal airway dimensions for Pugs and French Bulldogs with BOAS using CT under general anaesthesia [3]. In that paper, breed specific values for palate length and thickness were reported for both breeds, with comparisons to the skull index and body weight. Similar measurements were not performed in our population of dogs, as our population was heterogenous. The findings of this study suggest there is potential for future studies to perform such measurements on a bigger and homogenous population of Pugs and French Bulldogs undergoing conscious CT scanning to assess the impact of chemical immobilisation on the dimensions of the soft palate and nasopharynx. 

Our study also included four dogs that had previously undergone surgery for BOAS. Interpretation of the post-operative soft palate thickness in our population is challenging, because no information exists in the current literature about normal post-operative soft palate dimensions in awake dogs. However, the length of the soft palate was tomographically assessed as normal in all four dogs. Moderate thickening of the soft palate was recorded in one patient that had undergone a folded flap palatoplasty. Two patients in this group had a mild soft palate thickening, whereas one patient in this group had a normal thickness of the soft palate recorded. Two of the patients, one with a normal soft palate thickness and one with mild soft palate thickening, were Cavalier King Charles Spaniels, which in the authors’ experience rarely present with an excessive soft palate thickness. The remaining dog in the post-operative group had undergone two staphylectomy procedures, which had resulted in a normal soft palate length. It is unknown if the latter dog had been diagnosed with a thickened palate prior to these surgeries.

One notable concern in the context of upper airway imaging is the potential impact of chemical immobilisation on airway dimensions in veterinary patients. The effect of anaesthetic drugs on muscle tone have been documented in both human [27,28,29,30] and veterinary patients [31,32], however there is a knowledge gap in regards to how sedatives and anaesthetic drugs may change the upper airway dimensions in veterinary patients. Our decision to perform conscious CT scans was aimed to minimise the impact of chemical immobilisation on airway dimensions, and to limit the risk of anaesthesia in these compromised patients. Although specific measurements were not taken from our patient population, our reported image quality would allow such measurements to be conducted in future studies. Performing pre- and post-operative conscious CT scanning in brachycephalic dogs could also allow comparison of such measurements before and after multi-level airway surgery. 

Traditional practice involves sedation or general anaesthesia to ensure adequate patient positioning and minimal motion artefacts. With the introduction of multidetector CT with a fast acquisition time, the requirements for sedation in human paediatric patients have dropped dramatically [33,34,35,36,37]. Imaging techniques have also been optimised to provide high-quality images despite the lack of sedation [36,38]. In previous studies involving conscious CT scanning of veterinary patients, motion artefacts have been reported as absent or mild [17,18,19]. In our population, motion artefacts were only encountered in 3 of the 17 dogs, however these artefacts did not hinder the assessment of the upper airway dimensions. 

Seven dogs in our population were investigated for BOAS with no previous history of multi-level airway surgery. Successful diagnostic imaging acquisition was carried out in these patients with minimal impact on their respiratory function. The CT images provided a complete holistic assessment of all anatomical features of the upper airways known to be of significance to patients with BOAS. This assessment allowed the owners of the dogs in the current study to make better decisions about whether to pursue further upper airway examination under general anaesthesia and subsequent surgical intervention, or conservative management without having to undergo a potentially hazardous anaesthesia event. The authors believe that the addition of conscious CT scanning in this patient population is valuable for owner counselling. Furthermore, conscious CT scanning offers a time- and cost-efficient alternative to advanced imaging, potentially leading to more widespread adoption in veterinary medicine. 

Nevertheless, there are limitations to this study, including its retrospective nature and the small, heterogenous sample size. The lack of upper airway examinations under general anaesthesia to validate conscious CT findings, particularly in dogs that had previously undergone multi-level airway surgery, represents a limitation. The lack of this information is likely driven by the owners’ wish to avoid general anaesthesia, which is one of the benefits with the conscious CT scan procedure. Prospective studies are needed to investigate upper airway dimensions in awake brachycephalic dogs with BOAS before and after surgery to better understand the changes in dimensions following multi-level airway surgery.

Another important limitation of this study is related to the inherent challenges associated with imaging conscious animals, particularly in mitigating for factors such as swallowing, vocalisation, tongue positioning, head position and other motion artefacts. These variables may influence both image quality and interpretation, potentially leading to misidentification of anatomical structures or the misattribution of artifacts as pathology. This poses a particular challenge in the larynx. While the direct effects of phonation on laryngeal morphology in dogs remain largely unknown, studies in humans have demonstrated its influence [39,40]. Despite the fact that none of the dogs in our current study was observed to be vocalising during image acquisition, the potential impact of subtle, low-grade phonation on our population remains uncertain. Furthermore, the impact of head position on laryngopharyngeal dimensions has been evaluated in dogs, using fluoroscopy [41]. The results of this research showed that the head position has a significant impact on laryngopharyngeal dimensions. In comparison with a neutral head position, the nasopharyngeal luminal diameter decreases, and the soft palate thickness increases, with a flexed head position. Conversely, the nasopharyngeal luminal diameter increases with an extended head position. Interestingly, when comparing laryngopharyngeal dimensions between conscious and sedated patients with a neutral head position, the authors reported that sedation did not affect the airway dimensions in this population [41]. However, it is unknown if the findings of sedated, laterally recumbent healthy beagles can be extrapolated to our population of conscious dogs with upper airway disease, positioned in a standing or sternal position. Although visual monitoring of the head position was performed during image acquisition to ensure a neutral head position, an inevitable variability in head position between patients will exist, which may introduce the potential for inaccuracies in assessing laryngopharyngeal dimensions. 

Careful case selection and monitoring of the patients during image acquisition is important to reduce the chances of acquiring non-diagnostic images, which in turn will expose the dogs to unnecessary doses of ionising radiation, as well as unnecessary owner costs. Further investigation into the effects of phonation, head position and other behavioural factors on imaging outcomes in healthy conscious animals is warranted to enhance the validity and reliability of conscious CT scanning in veterinary medicine.

## 5. Conclusions

This study introduces the use of a 160-slice CT scanner for conscious image acquisition in dogs with upper airway disease. This is also the first study reporting conscious CT findings in dogs that had previously undergone multi-level airway surgery for BOAS. This research opens the door to future studies in this emerging field.

## Figures and Tables

**Table 1:** Clinical features of 17 dogs undergoing conscious computed tomography scans.

Case	Sex	Age (Months)	Breed	Condition	CT Findings	Direct Laryngoscopy Findings	Endoscopy Findings
1 ^†^	MN	19	French Bulldog	BOAS	Normal SP length. Moderate SP thickening. Open larynx.		
2 ^‡^	MN	17	Chihuahua	Persistent coughing	Normal SP length. Normal SP thickness. Open larynx.		
3 *	FS	64	Pug	BOAS	Overlong SP. Moderate SP thickening. Severe LC. Diffuse oesophageal gas dilation.		
4 ^‡^	FS	148	Cocker Spaniel	Nasopharyngeal narrowing	Normal SP length. Normal SP thickness. Open larynx. Resolution of nasopharyngeal narrowing. Unilateral otitis media.	Normal SP length. Normal SP thickness. Normal larynx. Everted tonsils.	
5 ^†^	FS	99	CKCS	BOAS	Normal SP length. Normal SP thickness. Open larynx.		
6 *	MN	13	Pug	BOAS	Overlong SP. Mild SP thickening Everted LS Aberrant caudal nasal conchae	Overlong SP. Thickened SP. Stage 1 LC. Everted tonsils.	
7 *	MN	15	French Bulldog	BOAS	Overlong SP. Marked SP thickening. Open larynx.	Overlong SP. Thickened SP. Normal larynx. Everted tonsils.	
8 ^‡^	FS	30	CKCS	Sleep apnoea	Overlong SP. Mild SP thickening. Open larynx.	Normal SP length. Normal SP thickness. Normal larynx. Everted tonsils.	
9 *	M	28	French Bulldog	BOAS	Overlong SP. Marked SP thickening. Everted LS. Unilateral otitis media.		
10 *	F	3	French Bulldog	BOAS	Overlong SP. Moderate SP thickening. Open larynx. Tracheal hypoplasia. Unilateral otitis media.	Overlong SP. Thickened SP. Stage 1 LC. Everted tonsils.	
11 *	MN	47	French Bulldog	BOAS	Overlong SP. Marked SP thickening. Open larynx.	Overlong SP. Thickened SP. Stage 1 LC. Everted tonsils	
12 ^‡^	FS	47	Maltese	Nasopharyngeal narrowing and tracheal collapse	Overlong SP. Mild SP thickening. Open larynx. Resolution of nasopharyngeal narrowing. Moderate tracheal collapse.	Normal SP length. Normal SP thickness. Normal larynx.	Resolution of nasopharyngeal narrowing. Grade 2 tracheal collapse.
13 ^‡^	MN	50	Mixed breed	Reversed sneezing	Overlong SP. Mild SP thickening. Open larynx.		
14^†^	FS	150	CKCS	BOAS	Normal SP length. Mild SP thickening. Open larynx.		
15 *	MN	57	Pug	BOAS	Overlong SP. Moderate SP thickening. Severe LC Aberrant caudal nasal conchae.	Overlong SP. Thickened SP. Stage 3 LC	
16 ^‡^	M	67	Pomeranian	Tracheal collapse	Normal SP length. Normal SP thickness. Open larynx. Severe tracheal collapse.	Normal SP length. Normal SP thickness. Normal larynx.	Grade 4 tracheal collapse.
17 ^†^	MN	104	French Bulldog	BOAS	Normal SP length. Mild SP thickening. Everted LS. Advanced pulmonary metastatic disease.		

BOAS brachycephalic obstructive airway syndrome, CKCS Cavalier King Charles Spaniel, F female, FS female spayed, M male, MN male neutered, SP soft palate, LC laryngeal collapse, LS laryngeal saccules. * Patients with untreated BOAS (group I). ^†^ Patients previously treated for BOAS (group II). ^‡^ Patients with respiratory disease other than BOAS (group III).

## Data Availability

The data that support the findings are contained within this article and are available from the corresponding author upon reasonable request.
